# Modeling body size evolution in Felidae under alternative phylogenetic hypotheses

**DOI:** 10.1590/S1415-47572009005000004

**Published:** 2009-01-10

**Authors:** José Alexandre Felizola Diniz-Filho, João Carlos Nabout

**Affiliations:** 1 Departamento de Biologia Geral, Instituto de Ciências Biológicas, Universidade Federal de Goiás, Goiânia, GOBrazil; 2 Programa de Doutorado em Ciências Ambientais, Universidade Federal de Goiás, Goiânia, GOBrazil

**Keywords:** autocorrelation, body size, Felidae, phylogenetic eigenvector regression, phylogenies

## Abstract

The use of phylogenetic comparative methods in ecological research has advanced during the last twenty years, mainly due to accurate phylogenetic reconstructions based on molecular data and computational and statistical advances. We used phylogenetic correlograms and phylogenetic eigenvector regression (PVR) to model body size evolution in 35 worldwide Felidae (Mammalia, Carnivora) species using two alternative phylogenies and published body size data. The purpose was not to contrast the phylogenetic hypotheses but to evaluate how analyses of body size evolution patterns can be affected by the phylogeny used for comparative analyses (CA). Both phylogenies produced a strong phylogenetic pattern, with closely related species having similar body sizes and the similarity decreasing with increasing distances in time. The PVR explained 65% to 67% of body size variation and all Moran's *I* values for the PVR residuals were non-significant, indicating that both these models explained phylogenetic structures in trait variation. Even though our results did not suggest that any phylogeny can be used for CA with the same power, or that “good” phylogenies are unnecessary for the correct interpretation of the evolutionary dynamics of ecological, biogeographical, physiological or behavioral patterns, it does suggest that developments in CA can, and indeed should, proceed without waiting for perfect and fully resolved phylogenies.

## Introduction

Phylogenetic comparative methods developed since the middle 1980's are now commonly applied in areas of biological research, such as ecology, physiology and behavior, to explain how phylogenetic patterns in the traits of species can be associated to adaptive evolution (Martins, 2000; Freckleton *et al.*, 2002). It is also well-known that species, or other taxonomic units, may not represent independent observations for statistical analyses such as regression and correlation (Felsenstein, 1985; Martins and Garland, 1991; Martins *et al.*, 2002). Hence, many different forms of comparative analyses have been developed to take into account the lack of independence among species due to phylogenetic relationships (*i.e.*, phylogenetic autocorrelation) and to correctly approximate the Type I errors of statistical analyses of correlation between traits or between traits and other components of environmental variation (Martins and Garland, 1991; Gittleman and Luh, 1992; Martins and Hansen, 1996; Martins *et al.*, 2002; Garland *et al.* 2005). Furthermore, some studies have suggested that incorporating phylogenetic structure into data analyses allowed a better understanding of the processes underlying ecological, behavioral and physiological data (Hansen and Martins, 1996; Diniz-Filho, 2001).

Mainly because of the improvements and popularization of DNA sequence techniques and other molecular markers, there has been a marked increase in the number of available phylogenies that can be used as a basis in comparative analysis (Pagel, 1999; see Felsenstein, 2004). More importantly for comparative analyses, there are now techniques that combine phylogenies from different sources and based on different data types, such as morphology, molecular data or behavior, to generate complete, or nearly complete, trees for very large taxonomic groups (supertrees) (Bininda-Emonds, 2004).

The first supertree was built for Primates (Purvis, 1995) and a nearly complete supertree for more than 95% of current mammal species has recently been published by Bininda-Emonds *et al.* (2007). However, the first complete supertree for all living species in a large taxa was generated for worldwide Carnivora (Bininda-Emonds *et al.* 1999) and not only encompassed all 271 living Carnivora species but was comparatively well resolved with a relatively small number of polytomies for most subclades and has been widely used in comparative analyses (Sechrest *et al.*, 2002.; Diniz-Filho and Tôrres, 2002; Tôrres and Diniz-Filho, 2004; Diniz-Filho, 2004; Diniz-Filho *et al.*, 2007). More recently, however, Johnson *et al.* (2006) proposed a fully resolved felid phylogeny derived from 22,789 base pairs from autosomal, X-linked, Y-linked and mitochondrial genes, with many important differences in relation to the widely used supertree.

It is important to consider that, despite of the fact that well-resolved phylogenies are the core of comparative analyses, there are still many uncertainties regarding the methods and data needed to reconstruct phylogenies and supertrees (Webb *et al.*, 2002). Thus, despite the increasing use of comparative methods (Carvalho *et al.*, 2005), it is always important to understand how modeling trait evolution from comparative methods are sensitive to errors and uncertainties regarding tree topology (Martins and Garland, 1991; Martins, 1996; Martins and Housworth, 2002) and other related problems, such as taxon sampling (Ackerly, 2000). It is, therefore, of paramount importance to evaluate how errors in phylogeny reconstruction may affect the results of comparative analyses, as when modeling trait evolution. In this paper we used phylogenetic correlograms and phylogenetic eigenvector regression (PVR) to model body size evolution in worldwide Felidae (Mammalia, Carnivora) under alternative phylogenies. Our purpose was not to establish the validity of any of the phylogenies, but rather to evaluate how analyses of patterns in body size evolution can be affected by choosing one of them as the basis for comparative analyses.

**Figure 1:**
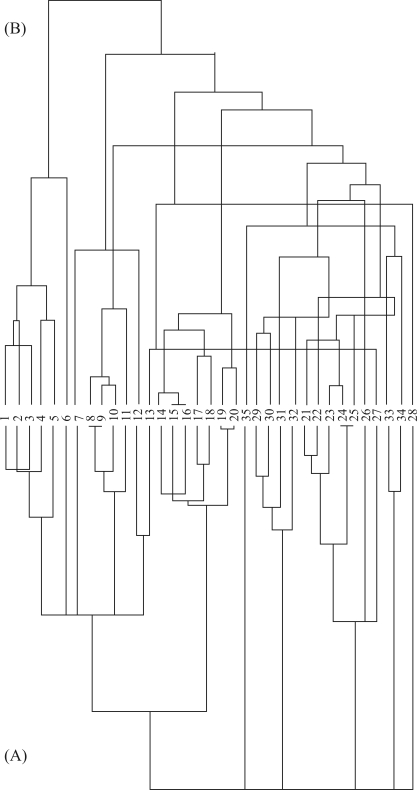
Phylogenies used in this study showing the relationships between the 35 felid species included in the analysis. In (A) the ST (Bininda-Emonds *et al.* (1999). Biol Rev 74:143-175) and (B) the JN with 10 (^†^Johnson et al. (2006). Science 311:73-77) phylogenies the numbers relate to the species shown in Table 1.

**Figure 2:**
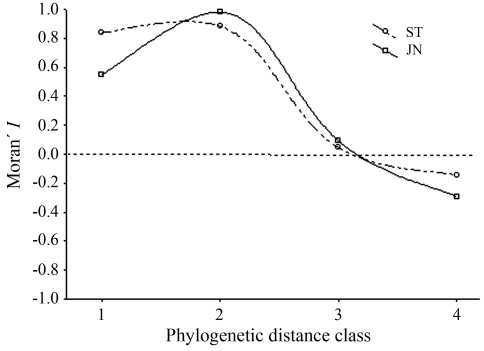
Phylogenetic correlograms for felid body size variation based on the phylogenies shown in Figure 1.

## Material and Methods

Body masses (in g) for 35 Felidae species (Table 1) distributed worldwide were obtained mainly from Smith *et al.* (2003). Values were transformed to decimal logarithms prior to the analysis. Because we were mainly interested in broad-scale comparative patterns, body sizes of males and females were averaged for each species. Also, although sexual dimorphisms exists for the group, the relatively magnitude of male-female differences when compared to interspecific differences across the entire clade is so small that this averaging is not likely to affect the conclusions reached in this paper.

We used the phylogenies proposed by Bininda-Emonds *et al.* (1999), designated the supertree (ST) phylogeny, and Johnson *et al.* (2006), designated the Johnson (JN) phylogeny, to establish pairwise phylogenetic distance matrices **D**, which were the basis for modeling body size evolution using Moran's *I* phylogenetic correlograms (Gittleman and Kot, 1990; Diniz-Filho, 2001) and phylogenetic eigenvector regression (Diniz-Filho *et al.*, 1998). For all analysis, the species *Catopuma badia*, *Felis catus*, *Felis ilbyca* and *Pardofelis badia* were eliminated because they were absent in both phylogenies. The two phylogenies have some important differences (Figure 1), with, for instance, the first divergence of felids occurring at 10.78 million years (My) in the JN phylogeny and at 16.2 My in the ST phylogeny. More relevant, however, were the differences in tree topology, with the ST phylogeny having many polytomies, and the two phylogenies also having some differences in the clades structure (Figure 1), with, for example, *Pathera* being basal to other clades in the JN phylogeny, which helps to explain some of the differences found later in the comparative analyses.

Moran's *I* is the most commonly used coefficient in spatial and phylogenetic autocorrelation analyses and allows the description of the phylogenetic structure of the data (or model residuals) and the comparison of the observed values with the expected values based on different evolutionary models (Gittleman and Kot, 1990; Diniz-Filho, 2001; Diniz-Filho and Tôrres, 2002). Moran Moran's *I* is given by


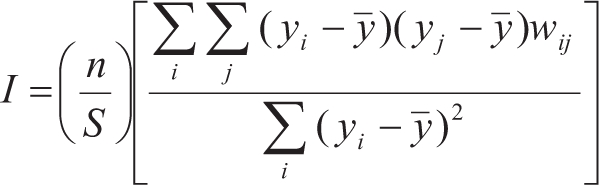


where *n* is the number of observation (species), *y*_*i*_ and *y*_*j*_ are the body size values in the species *i* and *j*, $\bar{y}$is the average of *y* and *w*_*ij*_ is an element of the matrix **W**. Actually, several Moran's *I* are calculated for the same variable, by creating successive **W** matrices in which *w*_*ij*_ = 1 if the pair *i*, *j* of species is within a given phylogenetic distance class interval, established based on **D** (indicating that species are “linked” in this class), and *w*_*ij*_ = 0 otherwise. In this paper, 4 classes with equal intervals (time slices) were used, and *S* indicates the number of entries (connections) in each **W** matrix. The value expected under the null hypothesis of the absence of spatial autocorrelation is -1/(*n* - 1). Moran's *I* usually vary between -1.0 for maximum negative autocorrelation and 1.0 for maximum positive autocorrelation. Computation details of the standard error of this coefficient (se(I)) are given in Legendre and Legendre (1998), so that Moran's *I* can be tested by assuming a normal distribution of se(I). In this case a standard normal deviate (SND = {I - E(I)}/se(I)) larger than 1.96 indicates that se(I) is significant at the 5% probability level (p = 0.05). The Bonferroni correction can be used for conservative statistical decisions, so that a correlogram will be considered significant at p < 0.05 only if one of Moran's *I* is significant at p = 0.05/4 distance classes.

It is also possible to use a direct modeling strategy to describe patterns of body size evolution. Diniz-Filho *et al.* (1998) developed a new technique called phylogenetic eigenvector regression (PVR) to partition the total variance (σ^2^_T_) of a trait **y** (*e.g.*, body size) measured in a set of species into phylogenetic (σ^2^_P_) and unique variances or ecological (σ^2^_S_) variances, such that σ^2^_T_**=** σ^2^_P_**+** σ^2^_S_. The idea being that a phylogeny can be expressed as a set of orthogonal vectors obtained by an eigenanalysis of a phylogenetic distance matrix **D**. These vectors can then be used as predictors of **y** in any form of linear or non-linear modeling. For analogous applications in a spatial context see Diniz-Filho and Bini (2005) and Griffith and Peres-Neto (2006). Thus, PVR follows the standard framework of general linear models, such that

**y** = **V**β + ε

where **V** is the orthogonal eigenvectors extracted from the double-centered phylogenetic distance matrix **D,** and β is the partial regression coefficients. The *R*^2^ value, adjusted to take into account a different number of predictors, of the multiple regression model of the trait **y** against the eigenvectors in **V** provides an estimate of the phylogenetic signal in the data (σ^2^_P_**/** σ^2^_T_).

There are different ways to establish how many eigenvectors of **D** should be used for modeling. In the work described in the present paper we used an exhaustive search strategy based on Akaike Information Criterion (AIC) described by Burnham and Anderson (2002). When performing the PVR, the AIC value of a particular model (*i.e.*, based on some combination of eigenvectors) was given by

AIC = *n* ln(σ^2^_S_) + 2*K*

where σ^2^_S_ is the variance of the previously defined specific component (PVR residuals) and *K* is the number of parameters (number of eigenvectors, plus the intercept and residual variance σ^2^_S_). The value of σ^2^_S_ can be used as a proxy for the likelihood of the model given the data, but this approximation is valid only if model errors are independent, normally distributed and with constant variance (Burnham and Anderson, 2002). The best models are those with the lowest AIC values but multiple PVR models can be generated, in which case a new spatial analysis in macroecology (SAM) module software (Rangel *et al.*, 2006; Diniz-Filho *et al.* 2008) was used to generate all possible combination of the first 10 eigenvectors from **D** as predictors, producing 1023 alternative models (*i.e.*, 2^*m*^ model minus the model with intercept only), allowing the establishment of the most parsimonious model for explaining body size variation based on phylogenetic eigenvectors. Moran's *I* correlograms of these PVR residuals were also applied to test if the model was effective in taking phylogenetic autocorrelation into account.

## Results

The correlograms based on the two phylogenies showed slightly different patterns, although there was a general trend of positive autocorrelation in the first two distance classes, with a larger value in the second class due to the more recent time slices, coupled with a negative autocorrelation in the last distance class (Figure 2; Table 2) (Diniz-Filho and Tôrres, 2002). In both phylogenies, closely related species have similar body sizes and this similarity decreases when increasing distances in time are considered, stabilizing in the last distance class (Table 2). However, despite the existence of a monotonic relationship between the two correlograms when using the JN tree there was a much lower Moran's *I* in the first distance classes, suggesting that there is some relatively high dissimilarity between more closely related species.

The proportion of variation explained by the phylogeny obtained from PVR is similar when based on the two phylogenies, despite the fact that the correlation between the first eigenvectors extracted from these matrices is relatively low (0.415). When based on the ST phylogeny, a model with 6 eigenvectors (summing 81.6% of the variation in original distance matrix) was selected by the AIC criterion as the minimum model, with R^2^ = 65.1% as compared with R^2^ = 69.1% for the full model with 10 eigenvectors. On the other hand, only three eigenvectors (explaining 45% of the total distances) were selected by AIC criterion to explain body size variation based on the JN phylogeny, and they explained 67.5% of the variation in body size as against R^2^ = 70.6% for the full model with 10 eigenvectors. In both cases, all Moran's *I* values for the PVR residuals were non-significant, indicating that these models were sufficient to explain phylogenetic structures in trait variation.

## Discussion

It is always important to check how results from comparative analyses of trait evolution are affected by errors or uncertainties in the phylogeny (Martins and Garland, 1991; Martins, 1996), when conflicting or alternative phylogenetic hypotheses are available for the group of organisms under scrutiny. Since the number of available phylogenies (including supertrees) is rapidly increasing in the literature, the simple solution is frequently to model trait evolution under these multiple phylogenies and compare the outcome of comparative analyses, as performed here.

Our analyses show that, despite the differences between the two phylogenies tested, phylogenetic autocorrelation patterns in body size of world felids revealed by correlograms and PVR were qualitatively similar. This suggests that previous general inferences about macroecological and macroevolutionary patterns using the Bininda-Emonds *et al.* (1999) supertree may be robust to changes in phylogeny, at least in terms of relative magnitude of phylogenetic patterns. For example, Diniz-Filho and Tôrres (2002) showed strong patterns in body size for New World Carnivora, using phylogenetic eigenvector regression and a significant correlation between body size and geographic range size, whereas Diniz-Filho *et al.* (2007) recently used PVR to decouple adaptive and historical components of Bergmann`s rule (*i.e.*, increase in body size towards higher latitudes) in European Carnivora. The correlogram shape and PVR results obtained in these studies for different subsets of Carnivora are qualitatively similar to the those obtained by us for felids alone.

Indeed, many papers have reported relatively strong phylogenetic patterns of body size variation between species, so this is probably due to real processes that generate strong phylogenetic inertia in this trait, almost independently of taxonomic scale and resolution. Hence, changes in phylogeny are unlikely to affect the estimate of this pattern too much, at least using statistically-based techniques such as PVR and correlograms. Some more general studies have shown consistent patterns regarding the phylogenetic structuring of different traits with, for example, morphological traits usually being more phylogenetically structured than behavioral or ecological traits (Freckleton *et al.*, 2002; Morales, 2000; see also Carvalho *et al.*, 2005). Since these observations were undertaken in a meta-analytical context, involving many different clades from different regions of the world, they are unlike to have been strongly affected by changes in the particular phylogenies used in the analyses.

It is difficult to generalize the results described in this paper, although it is possible to evaluate why statistical methods used here would work fine and provide similar results under alternative phylogenies. Because eigenanalysis generates a hierarchical structure of representation of phylogenetic patterns of relationship from the root to the tips of the phylogeny, PVR is probably less sensitive to errors in phylogeny that presumably tend to occur in more recent branches (Diniz-Filho *et al.*, 1998). If discussions about the validity of the relationship are mainly focused on recent nodes with less clear signals, there will be reasonable estimates using different trees if trait evolution is stationary. Even so, in our analyses there were some important differences in deep branches (such as the relationship between *Panthera* and other subclades), although the correlogram suggests that, for both phylogenies, covariance in body size has been stronger in recent times, probably due to non-linear components (stabilizing selection) involved in body size evolution (Diniz-Filho, 2004). Correlograms may also be not very sensitive when using large distance classes between time slices, as used here, because of the small number of species. Even if there are different phylogenies, it is likely that most of the pairwise comparisons fall within the same distance classes, creating similarity between correlograms.

However, other patterns detected from phylogenies may be more sensitive to errors and uncertainties and should be tested in future studies. For instance, estimation of phylogenetic diversity, based on summing branch lengths or the ages of the most recent common ancestors (Sechrest *et al.*, 2002; Diniz-Filho, 2004; Tôrres and Diniz-Filho, 2004) may be more sensitive to changes in phylogeny since assemblage compositions will be considered independently (reducing sample sizes) so that errors would be magnified at these smaller geographical scales.

It is worth noting that our main conclusions do not mean that any phylogeny, independently of the resolution, can be used in comparative analysis with the same power, or that “good” phylogenies are not necessary to give the correct interpretations to the evolutionary dynamics of ecological, biogeographical, physiological or behavioral patterns. However, results such as those presented in this paper support the view that developments in comparative analysis in many fields of biology can, and indeed must, occur within the context of dealing with less-than-perfect or not fully resolved phylogenies.

## Figures and Tables

**Table 1:** Felid species for the Supertree (ST)* and Johnson (JN)^†^ phylogenies. The scientific names are mostly the same in both the phylogenies, the exceptions being that the genus names in parentheses are as given in the JN phylogeny. Common names and rounded mean body mass values are also given.

Code	Scientific name	Common name	Approximate mean body mass (kg)^-^
1	*Panthera leo*	Lion	159
2	*Panthera pardus*	Leopard	52
3	*Panthera onca*	Jaguar	85
4	*Panthera tigris*	Tiger	163
5	*Uncia uncia*	Snow leopard	33
6	*Neofelis nebulosa*	Clouded leopard	15
7	*Pardofelis marmorata*	Marbled cat	3
8	*Lynx canadensis*	Canadian lynx	10
9	*Lynx lynx*	Eurasian lynx	19
10	*Lynx pardinus*	Iberian, or Spanish, lynx	11
11	*Lynx rufus*	Bobcat	27
12	*Catopuma* (*Pardofelis*) *temminckii*	Asian golden cat	8
13	*Profelis* (*Caracal*) *aurata*	African golden cat	11
14	*Leopardus tigrinus*	Oncilla or little spotted cat	2
15	*Oncifelis geoffroyi*	Geoffroy's cat	3
16	*Oncifelis guigna*	Kodkod	3
17	*Oncifelis colocolo*	Pampas cat	4
18	*Oreailurus jacobita*	Andean mountain cat	8
19	*Leopardus pardalis*	Ocelot or painted leopard	12
20	*Leopardus wiedii*	Margay	3
21	*Felis margarita*	Sand cat	3
22	*Felis nigripes*	Black-footed cat	1
23	*Felis silvestris*	Wildcat	5
24	*Felis bieti*	Chinese mountain cat	6
25	*Felis chaus*	Jungle cat	7
26	*Otocolobus manul*	Pallas, or steppe, cat	3
27	*Caracal caracal*	African, or Persian, lynx	12
28	*Leptailurus* (*Caracal*) *serval*	Serval	12
29	*Prionailurus bengalensis*	Leopard cat	3
30	*Prionailurus viverrinus*	Fishing cat	9
31	*Prionailurus rubiginosus*	Rusty spotted cat	1
32	*Prionailurus planiceps*	Flat-headed cat	4
33	*Herpailurus* (*Puma*) *yagouaroundi*	Jaguarundi cat	7
34	*Puma concolor*	Cougar	54
35	*Acinonyx jubatus*	Cheetah	51

^*^Bininda-Emonds *et al.* (1999). ^†^Johnson *et al.* (2006). ^-^Mainly from Smith *et al.* (2003).

**Table 2:** Moran's *I*, standard error (SE) and standard normal deviate (SND) obtained for body size and the phylogenetic eigenvector regression (PVR) residual for each phylogeny used in this study.

	Supertree (ST) phylogeny				Johnson (JN) phylogeny		
	Moran's I	SE	SND		Moran's I	SE	SND
Body size							
1	0.840	0.142	6.140***		0.550	0.209	2.770**
2	0.882	0.253	3.605**		0.979	0.153	6.597***
3	0.046	0.092	0.820		0.093	0.036	3.436**
4	-0.144	0.020	-5.882***		-0.292	0.027	-9.753

PVR residuals							
1	-0.196	0.143	-1.170		-0.013	0.211	0.078
2	-0.251	0.255	-0.868		0.221	0.155	1.618
3	-0.066	0.093	-0.391		-0.094	0.036	-1.817
4	-0.003	0.020	1.361		-0.011	0.027	0.682

**p < 0.01; ***p < 0.001.
